# 液液萃取结合超高效液相色谱-串联质谱同时测定尿中5种常见拟除虫菊酯代谢产物

**DOI:** 10.3724/SP.J.1123.2025.10017

**Published:** 2026-07-08

**Authors:** Xiaomei ZHANG, Xiangjuan MENG, Yue HU, Xiaodong LIU

**Affiliations:** 北京市职业病防治院中心实验室，北京 100176; Central Laboratory，Beijing Prevention and Treatment Hospital of Occupational Disease，Beijing 100176，China

**Keywords:** 尿样, 拟除虫菊酯, 代谢产物, 超高效液相色谱-串联质谱, 液液萃取, urine, pyrethroids （PYRs）, metabolites, ultra performance liquid chromatography-tandem mass spectrometry （UPLC-MS/MS）, liquid-liquid extraction （LLE）

## Abstract

拟除虫菊酯广泛应用于农业病虫害防治及家庭卫生消杀等领域，可对人体产生多种健康损害。建立准确、灵敏、高效的生物监测方法，评估拟除虫菊酯在不同人群的内暴露水平，已成为环境暴露与健康效应研究的一项重要课题。本研究通过盐酸水解和液液萃取对样本进行前处理，建立了超高效液相色谱-串联质谱同时测定尿中5种常见拟除虫菊酯代谢产物的分析方法。尿样经盐酸水解，乙酸乙酯萃取，BEH C_18_色谱柱分离，在负离子模式、多反应监测条件下扫描质谱信息，采用工作曲线和内标法定量分析**。**结果表明，5种拟除虫菊酯代谢产物线性关系良好，工作曲线的相关系数均大于0.995，检出限为0.13~1.32 ng/mL，定量限为0.44~4.39 ng/mL。样品在20、50、80 ng/mL 3个加标水平下的平均回收率为91.0%~102.0%，批内精密度为1.1%~8.1%，批间精密度为1.1%~4.6%，样本在4 ℃下可稳定一周。本方法样品前处理操作简便高效，经济适用性强，方法检出限低，准确度和精密度高，可为不同人群，尤其是一般居民、孕妇、儿童等敏感群体的拟除虫菊酯暴露评估提供技术参考。

## Abstract (en)

Pyrethroids are extensively employed in agricultural pest management and household sanitation practices. However， their widespread use has raised concerns as they pose a multitude of health risks to humans. Consequently， the development of precise， highly sensitive， and efficient biomonitoring techniques for evaluating internal exposure levels of pyrethroids across different populations has emerged as a paramount goal in the field of environmental exposure and health effect research. This study developed a method using ultra performance liquid chromatography-tandem mass spectrometry （UPLC-MS/MS） for the simultaneous determination of five common pyrethroid metabolites in urine， with sample pretreatment involving hydrochloric acid hydrolysis and liquid-liquid extraction. The method was optimized for mass spectrometric acquisition parameters and liquid chromatography separation conditions. Chromatographic separation was successfully accomplished utilizing a BEH C_18_ column （100 mm×2.1 mm，1.7 μm）. Mass spectrometric data were acquired in negative ion mode under multiple reaction monitoring （MRM） conditions. Among the analytes， *trans*-3-（2，2-dichlorovinyl）-2，2-dimethylcyclopropanecarboxylic acid （*trans*-DCCA） and *cis*-3-（2，2-dichlorovinyl）-2，2-dimethylcyclopropanecarboxylic acid （*cis*-DCCA） are isomers， as are 2-phenoxybenzoic acid （2-PBA） and 3-phenoxybenzoic acid （3-PBA）， and they share identical MS acquisition parameters. Identification was based on reference standards and retention times. The mobile phase consisted of 0.1% acetic acid in water （A） and acetonitrile （B）. The gradient elution program was as follows： 0-0.5 min， 10%B； 0.5-4.5 min， 10%B-70%B； 4.5-5 min， 70%B-100%B； 5-7 min， 100%B； 7-8 min， 100%B-10%B； 8-10 min， 10%B. The optimization of urine sample pretreatment conditions was divided into two parts： hydrolysis and extraction. Using the recoveries of target analytes as the evaluation metric， parameters including the dosage of hydrolysis reagent， hydrolysis temperature and duration， as well as the type， dosage， and extraction time of the extraction solvent were systematically optimized. The optimized pretreatment protocol is delineated as follows： Initially， 40 µL of the 2-PBA internal standard working solution was precisely added to 1 mL of urine sample， followed by thorough mixing to ensure homogeneity. Subsequently， 150 µL of hydrochloric acid （2 mol/L） was introduced to facilitate hydrolysis， which was allowed to proceed at ambient room temperature for a duration of 30 min. Finally， extraction was carried out using 2 mL of ethyl acetate， accompanied by vigorous shaking for 30 min to maximize extraction efficiency. Following centrifugation， the organic phase was separated， evaporated to near dryness， and reconstituted with 1.0 mL of acetonitrile prior to instrumental analysis. The matrix effects were evaluated using pure solvent and matrix-matched standards. Among the five analytes， three exhibited a moderate matrix effect， while two showed a weak matrix effect. The recovery performance using 2-PBA as an internal standard was also assessed. Consequently， a combination of the working curve and the internal standard method was selected for the quantification of the target analytes. Based on this， the methodological parameters of the method were validated. The results indicate that the five pyrethroid metabolites exhibited good linearity， with correlation coefficients of the calibration curves all exceeding 0.995. The limits of detection （LODs） ranged from 0.13 ng/mL to 1.32 ng/mL， and the limits of quantification （LOQs） ranged from 0.44 ng/mL to 4.39 ng/mL. The average recoveries of the samples at three spiked levels of 20， 50， and 80 ng/mL ranged from 91.0% to 102.0%. The intra-batch precision was between 1.1% and 8.1%， while the inter-batch precision was between 1.1% and 4.6%. Sample stability was demonstrated for at least one week when stored at 4 ℃. The established method was applied to analyze 18 urine samples from the general population. Neither *cis*-DCCA nor 4-fluoro-3-phenoxybenzoic acid （4F-3PBA） was detected in any sample. The mass concentration of 3-PBA ranged from 0.69 ng/mL to 1.59 ng/mL， with a detection rate as high as 88.9%. These results are largely consistent with screening studies on human pyrethroid metabolite levels reported in domestic and international literature in terms of both the detection rate and mass concentration range of 3-PBA. The presence of 3-PBA may originate from household insecticide exposure or dietary sources. The method demonstrates simple and efficient sample pretreatment， strong cost-effectiveness， low LODs， and high accuracy and precision. It can therefore serve as a reliable technical reference for monitoring and exposure assessment in various populations， particularly sensitive groups such as the general population， pregnant women， and children.

拟除虫菊酯（pyrethroids， PYRs）是一类应用广泛的人工合成杀虫剂，人体主要通过食物、水体、空气及皮肤接触等途径暴露于此类物质^［1-3］^。农业劳动者、病虫害防治员、畜牧业者、生产工人及应急响应人员等是其主要职业暴露群体，此外，PYRs也常用于家居环境，使用场景多样。早期研究认为拟除虫菊酯在哺乳动物体内代谢迅速且毒性较低，但随着流行病学证据的积累，其暴露已被证实可能与多种健康损害相关，包括生殖系统损伤^［4］^、免疫功能影响^［5］^、干扰内分泌^［6，7］^及神经毒性^［8，9］^等，尤其对儿童神经发育和肺功能的不良影响已引发广泛关注^［10-12］^。这些发现推动了对拟除虫菊酯毒性机制的重新审视，并促进了相关健康防护策略的深入研究。PYRs的人体代谢主要为氧化和水解反应，多数代谢物易与葡萄糖醛酸、氨基酸等内源性物质结合产生水溶性代谢产物，随尿排出^［13］^。常见PYRs的代谢产物主要有5种，分别为顺式-3-（2，2-二氯乙烯基）-2，2-二甲基环丙烷-1-羧酸（*cis*-DCCA）、反式-3-（2，2-二氯乙烯基）-2，2-二甲基环丙烷-1-羧酸（*trans*-DCCA）、3-苯氧基苯甲酸（3-PBA）、4-氟-3-苯氧基苯甲酸（4F-3PBA）及顺-3-（2，2-二溴乙烯基）-2，2-二甲基环丙烷-1-羧酸（*cis*-DBCA），其中4F-3PBA仅来源于氰戊菊酯的特异性代谢；*cis*-DBCA仅由溴氰菊酯特异性代谢产生；*cis*-DCCA可由氯菊酯、氯氰菊酯、溴氰菊酯、氟氯氰菊酯代谢生成；*trans*-DCCA源于氯菊酯或氯氰菊酯的代谢；3-PBA是大多数拟除虫菊酯类农药（如氯氰菊酯、高效氯氟氰菊酯、氯菊酯、联苯菊酯、氟氯氰菊酯、甲氰菊酯、溴氰菊酯等）在生物体内共有的特异性代谢产物^［14］^，目前未有相关文献指出人体会因接触其他有害物质代谢生成3-PBA，在实际监测中，3-PBA既可单独用于评估人群对该类农药的总暴露水平，亦可结合其他特征代谢产物以区分具体农药的接触类型^［15］^。拟除虫菊酯暴露生物监测中常用的生物样本有血清和血浆^［16，17］^、尿液^［18］^、唾液^［19］^及汗液^［20］^等，其中尿样因其无创、易采集的特性，在大规模人群研究中使用最广泛。现有检测方法包括气相色谱-串联质谱、液相色谱及液相色谱-串联质谱等，但普遍存在代谢物分析种类覆盖不足、前处理复杂、成本高昂等问题。例如，卢大胜等^［21］^基于气相色谱-质谱联用技术建立了尿中3种拟除虫菊酯代谢产物的检测方法，样品经盐酸水解后正己烷提取，再以硫酸-甲醇进行甲酯化衍生后检测，处理成本低，但需衍生提高灵敏度。孟潇等^［22］^将尿样水解后经固相萃取以液相色谱测定3-PBA，检出限和定量限均较高，无法满足普通人群监测需求；陈玉婕^［23］^和张续等^［15］^通过酶解和固相萃取结合液相色谱-质谱联用技术分别实现了对尿中4种和3种拟除虫菊酯代谢物的分析，但样品酶解均需过夜，效率较低，且固相萃取涉及淋洗、洗脱、氮吹、复溶等一系列操作致使分析物损失率较大，需引入同位素内标校正回收率；固相萃取柱、同位素标准品及水解酶的高昂价格严重限制了方法的推广应用。针对上述问题，本研究通过建立酸水解结合液液萃取的超高效液相色谱-串联质谱（UPLC-MS/MS）分析方法，实现了尿液中5种拟除虫菊酯代谢物的同时测定。相比已有研究，本方法检测种类较为全面，样本前处理简便高效、成本较低且结果精密度高，适用于大范围人群筛查，可为不同拟除虫菊酯接触人群，尤其是一般居民、孕妇、儿童等敏感群体及职业暴露监测研究提供技术参考。

## 1 实验部分

### 1.1 仪器与试剂

UPLC I-Class Xevo TQ-XS超高效液相色谱-串联质谱仪（美国Waters公司）；SQP十万分之一天平（德国Sartorius公司）；Vortex-Genie^®^ 2涡旋振荡器（美国Scientific Industries公司）；SCDEALL VX-Ⅱ 多管涡旋振荡器（北京安简科技有限公司）；CH210R高速离心机（湖南湘仪实验室仪器开发有限公司）；Concentrator plus^TM^真空浓缩仪（德国Eppendorf公司）。

*cis*-DCCA（1 000 mg/L，溶剂为乙腈）、*trans*-DCCA（1 000 mg/L，溶剂为甲醇）、4F-3PBA标准溶液（100 mg/L，溶剂为乙腈）均购自上海安谱实验科技股份有限公司；*cis*-DBCA（100 mg/L，溶剂为甲醇）、3-PBA标准溶液（100 mg/L，溶剂为甲醇）均购自天津阿尔塔科技股份有限公司；2-苯氧基苯甲酸（2-phenoxybenzoic acid，2-PBA，≥99.0%）、甲酸（质谱级）购自日本TCI公司；乙酸铵（质谱级）购自上海安谱实验科技股份有限公司；乙酸、甲醇、乙腈均为质谱级，购自美国Fisher公司；蒸馏水购自广州屈臣氏公司。

### 1.2 溶液配制

将5种标准品用乙腈配制成1 000 ng/mL的混合标准使用液。准确称取10.0 mg 2-PBA标准品，用乙腈配制成500 ng/mL的2-PBA内标使用液。采用非接触者混合尿液为基质，通过加入不同体积的混合标准使用液制备质量浓度分别为0、1、5、10、30、60、80、100 ng/mL的系列标准工作液。

### 1.3 样品前处理与测定

取1.0 mL尿样，加入40 μL 2-PBA内标使用液（500 ng/mL）混匀，再加入150 μL盐酸（2 mol/L）室温下水解30 min，以2 mL乙酸乙酯振荡萃取30 min，离心后分离有机相蒸发至近干，用1.0 mL乙腈复溶后进行测定。

本研究经北京市职业病防治研究院伦理委员会批准，编号为C2025021。

### 1.4 实验条件

#### 1.4.1 色谱条件

色谱柱：ACQUITY UPLC BEH C_18_色谱柱（100 mm×2.1 mm，1.7 μm，美国Waters公司）；柱温：40 ℃；进样量：5 μL；流动相A：0.1%乙酸水溶液；B：乙腈；流速：0.2 mL/min。洗脱程序：0~0.5 min，10%B；0.5~4.5 min，10%B~70%B；4.5~5 min，70%B~100%B；5~7 min，100%B；7~8 min，100%B~10%B；8~10 min，10%B。

#### 1.4.2 质谱条件

电喷雾离子源，负电离模式，多反应监测（MRM）模式扫描，毛细管电压：1.0 kV；离子源温度：150 ℃；脱溶剂气温度：450 ℃；脱溶剂气流量：900 L/h；锥孔气流量：150 L/h，质谱采集参数见表1。

**表1:** 5种分析物和内标的质谱采集参数

No.	Compound	Abbr.	Parent ion （*m/z*）	Product ions （*m/z*）	Cone voltage/V	Collision energies/eV	*t*_R_/min
1	*trans*-3-（2，2-dichlorovinyl）-2，2-dimethylcyclopropanecarboxylic acid	*trans*-DCCA	206.9	35.0^*^， 119.0	30	12， 10	5.53
2	*cis*-3-（2，2-dichlorovinyl）-2，2-dimethylcyclopropanecarboxylic acid	*cis*-DCCA	206.9	35.0^*^， 119.0	30	12， 10	5.72
3	3-phenoxybenzoic acid	3-PBA	213.1	169.0^*^， 145.0	20	12， 15	5.51
4	4-fluoro-3-phenoxybenzoic acid	4F-3PBA	230.9	92.9^*^， 187.0	22	22， 8	5.55
5	（1*R*-*cis*）-3-（2，2-dibromoethenyl）-2，2-dimethylcyclopropane carboxylic acid	*cis*-DBCA	296.8	80.9^*^， 183.0	30	20， 12	5.85
6	2-phenoxybenzoic acid^#^	2-PBA	213.1	169.0^*^， 145.0	20	12， 15	5.19

* Quantitative ion. # IS.

## 2 结果与讨论

### 2.1 质谱采集参数

配制50 ng/mL的标准溶液，通过质谱直接进样，观察不同采集模式下母离子的信号强度，调节毛细管电压、脱溶剂气温度、流量等参数，进行子离子扫描，以丰度最高的特征碎片离子作为定量子离子，手动优化锥孔电压和碰撞能；*trans*-DCCA与*cis*-DCCA、2-PBA与3-PBA为两组同分异构体，质谱采集参数相同，以标准品和保留时间定性；具体质谱参数见表1。

### 2.2 液相色谱分离条件

本研究通过控制变量法，以色谱峰形、响应强度、分离效果为主要评价指标，系统优化了色谱柱类型、流动相组成、梯度洗脱程序及流速等液相色谱条件。在色谱柱选择方面，比较了ACQUITY UPLC HSS T_3_（100 mm×2.1 mm，1.8 μm，美国Waters公司）与ACQUITY UPLC BEH C_18_两种常用色谱柱的性能。两者均能实现对目标化合物的有效分离，但C_18_柱在响应强度方面表现更优，尤其对仪器响应较弱的*cis*-DCCA和*cis*-DBCA两种化合物灵敏度提升更为明显。因此，后续实验选择BEH C_18_色谱柱进行分析。对比多种水相与有机相组合优化流动相组成，结果表明：与相同体积分数的甲酸相比，乙酸作为水相添加剂能显著提升多数分析物的响应强度；但当乙酸体积分数从0.1%提高至0.2%时，整体响应呈下降趋势。使用乙酸铵溶液作为水相时，响应明显低于甲酸或乙酸体系。此外，在有机相（甲醇或乙腈）中添加甲酸或乙酸会导致响应降低。综合考量，最终选择0.1%乙酸水溶液与乙腈作为流动相体系。根据分析物出峰时的流动相比例优化梯度洗脱程序，使其更匹配目标物的保留特性。在流速优化时，考察了0.1~0.3 mL/min 流速下目标物的分离与响应变化。随流速增大，峰面积逐渐下降；在0.1 mL/min条件下，*cis*-DCCA与*trans*-DCCA分离度不足；0.15 mL/min时虽能实现基线分离，但在实际样本中3-PBA与基质干扰峰分离不理想。最终选择0.2 mL/min作为分析流速，该条件下5种目标物及内标2-PBA均能实现完全分离，峰形良好。图1为在优化条件下获得的加标尿样典型色谱图。

**图1:**
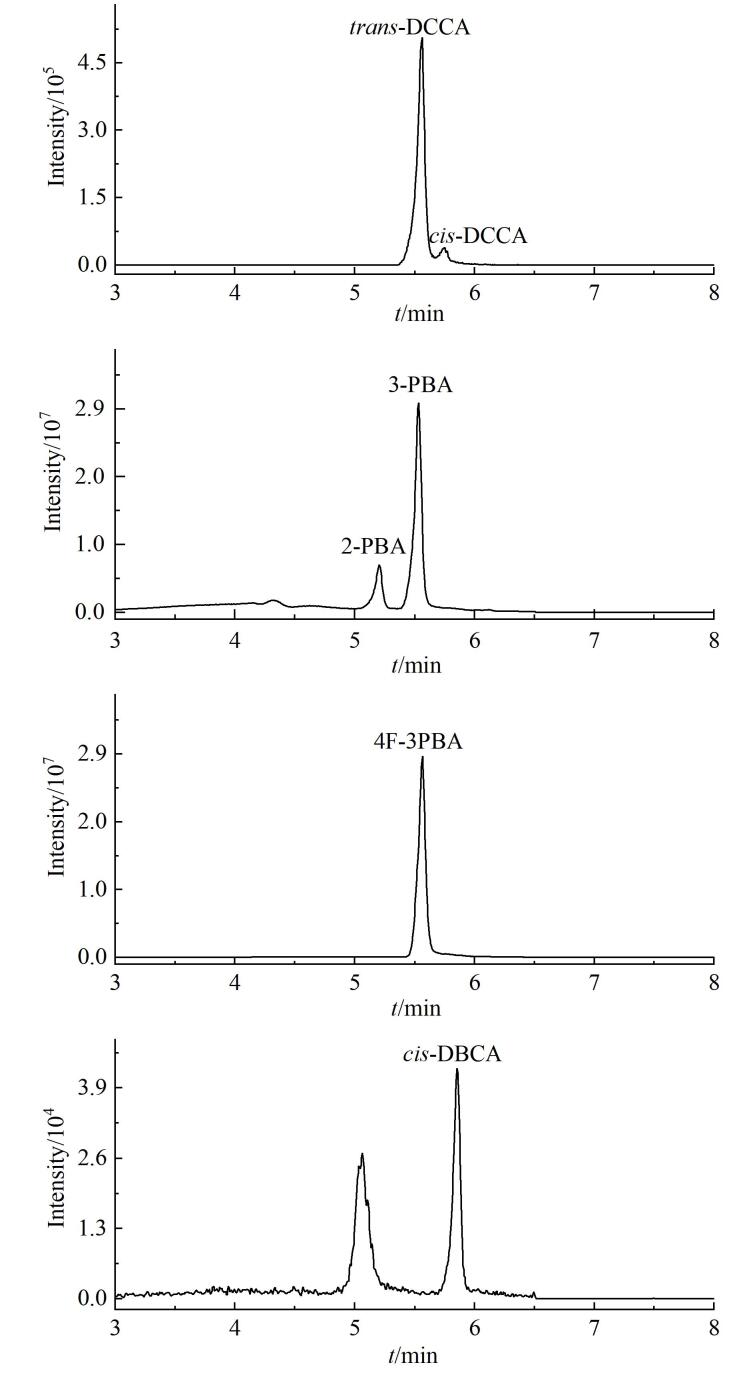
非职业接触人群加标样品中5种分析物与内标的提取离子色谱图

### 2.3 尿样前处理条件优化

尿样的前处理流程主要包括水解与萃取两个关键步骤。以非接触者混合加标尿样为基质，采用控制变量法，以加标回收率为评价指标，评估前处理条件对目标物提取效率的影响。

#### 2.3.1 水解条件

尿中拟除虫菊酯代谢物的盐酸水解，是通过酸裂解化学键使结合态转变为游离态，提升在有机溶剂中的可萃取性。本研究以加标水平为50 ng/mL的非接触者混合尿样为对象，系统考察了盐酸添加量、水解温度和时间3个关键参数，结果如图2所示。在盐酸添加量优化中，当2 mol/L盐酸体积由20 μL增至200 μL时，*trans*-DCCA的回收率呈现先上升后下降的趋势，并于150 μL时达到峰值。该峰值点与100 μL的回收率差异极显著（*p*<0.01），而与200 μL时差异不显著（*p*>0.05）。其余4种分析物在不同盐酸添加量下的回收率均无统计学差异（*p*>0.05）。因此，确定最佳盐酸添加量为150 μL。就水解温度而言，考察了室温（room temperature， RT，约20~25 ℃）、50、80及100 ℃ 4个水平。随着温度升高，5种分析物的回收率整体呈下降趋势。其中，室温与50 ℃条件下的回收率差异不显著（*p*>0.05），80 ℃与100 ℃之间亦无显著差异（*p*>0.05）。鉴于室温下水解已可获得满意回收率，且操作简便、节能，故选择室温作为水解温度。水解时间的影响表现为回收率随时间的延长先升高后降低。特别地，*cis*-DBCA在10 min与20 min水解时间下的回收率差异极显著（*p*<0.01），其余各时间点间差异均不显著（*p*>0.05）。考虑到*cis*-DCCA与*cis*-DBCA的仪器响应灵敏度较低，且二者均在30 min时回收率达到最高，综合平衡效率与灵敏度，最终选取30 min为适宜水解时间。综上，本研究优化的尿样前处理水解条件确定如下：在1.0 mL尿样中加入150 μL 2 mol/L盐酸，于室温下反应30 min。

**图2:**
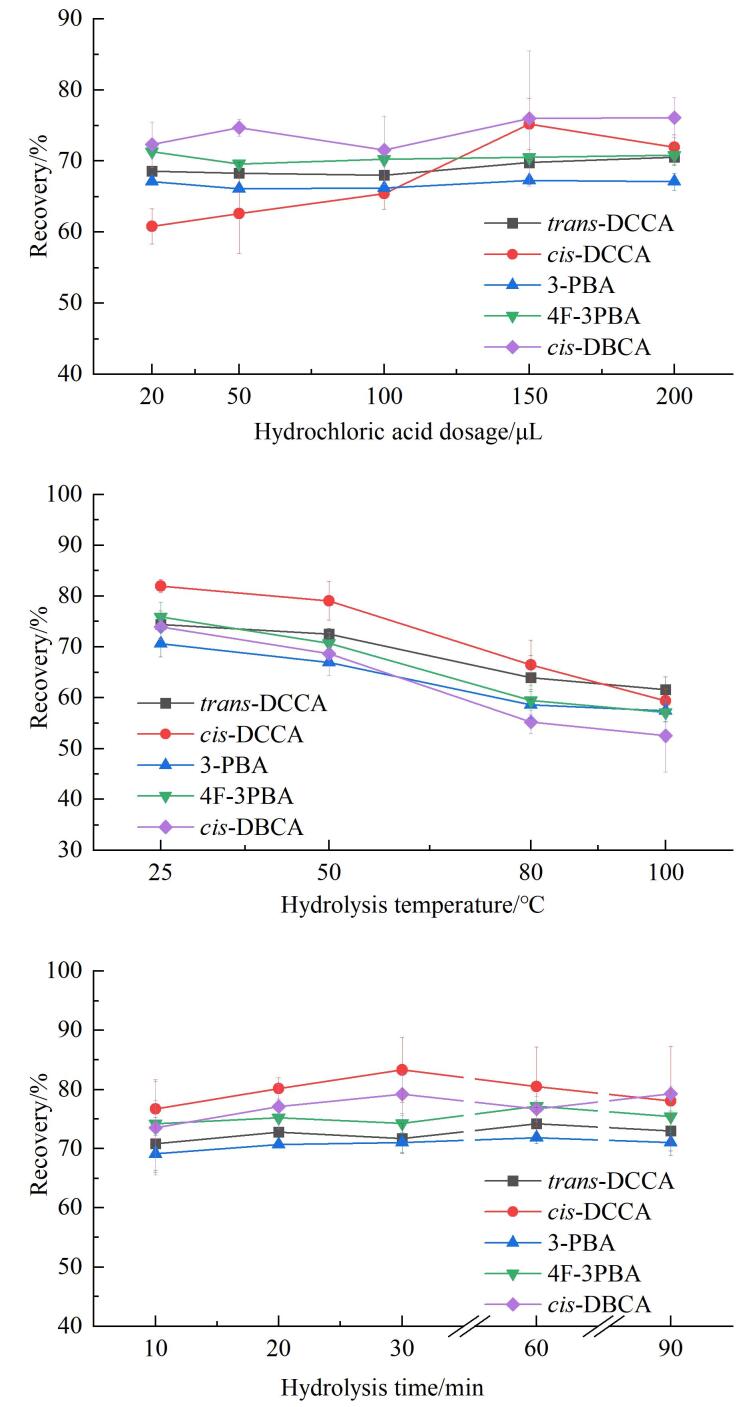
不同水解条件对5种分析物回收率的影响（*n*=3）

#### 2.3.2 萃取条件

采用优化后的水解条件处理50 ng/mL加标尿样，系统考察了二氯甲烷、乙酸乙酯、乙醚及正己烷4种不同极性溶剂对目标分析物的萃取性能（图3）。结果表明，4种溶剂的萃取回收率范围为47.7%~63.6%，其中乙酸乙酯的回收率精密度最佳（RSD<10%），优于其他溶剂。酸性条件下直接萃取回收率更高，而添加氯化钠使回收率下降7.0%~12.4%。因此，采用离心有效破除乳化并选用乙酸乙酯为萃取剂，省去中和与盐析步骤。随后优化了该萃取剂的用量及萃取时间。结果显示，当萃取剂用量在2~4 mL范围内，各分析物回收率无显著差异（*p*>0.05），故选择2 mL作为经济有效用量。延长萃取时间对各分析物回收率无显著影响，但30 min时多数分析物回收率相对较高且稳定性较好。最终确定萃取条件为2 mL乙酸乙酯，萃取30 min。

**图3:**
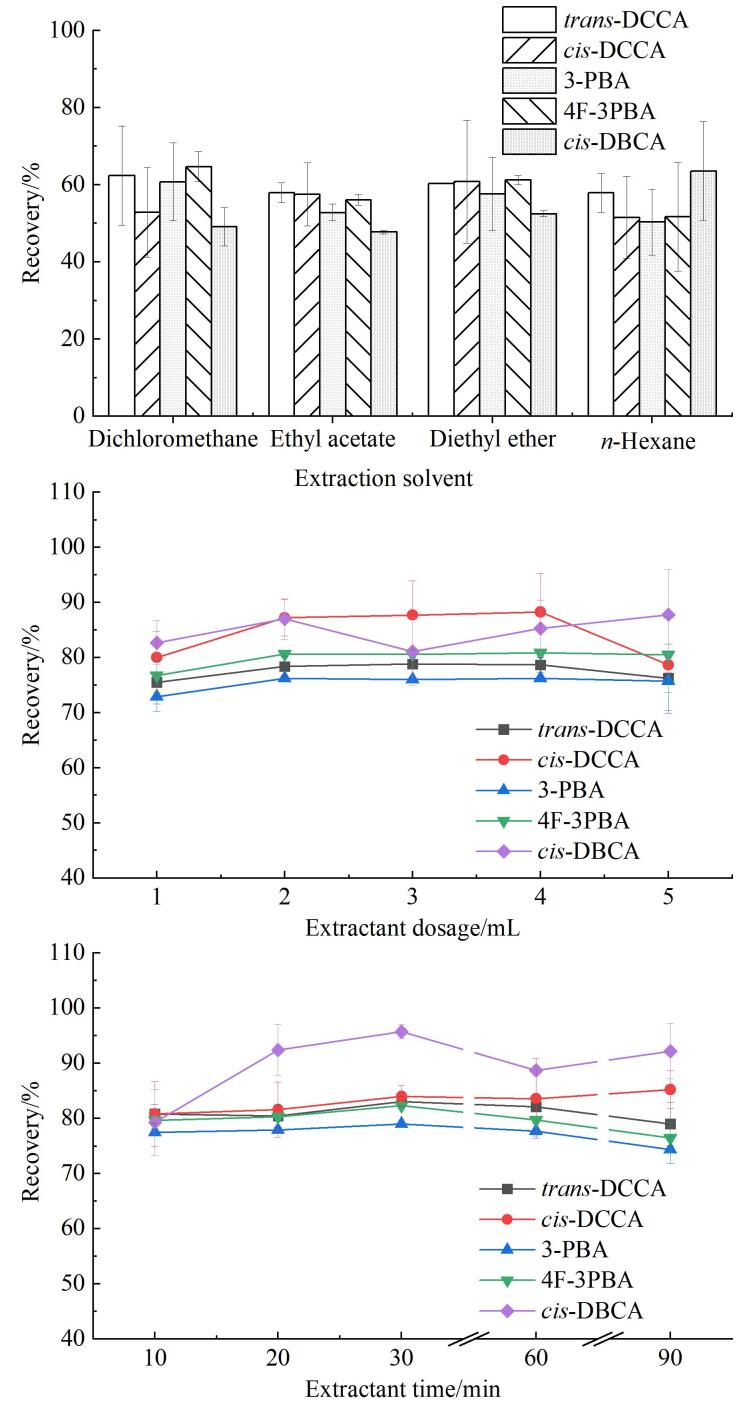
萃取剂种类、用量及萃取时间对5种分析物回收率的影响（*n*=3）

### 2.4 方法性能指标

#### 2.4.1 基质效应

本研究比较了以乙腈为溶剂的溶剂工作曲线与以非接触者混合尿液稀释制作的加标工作曲线的斜率比值，以评价基质效应：比值在80%~120%为弱基质效应，50%~80%或120%~150%为中等基质效应，<50%或>150%为强基质效应^［15］^。结果显示，*trans*-DCCA、3-PBA与4F-3PBA的基质效应比值分别为76.4%、72.0%和78.1%，属于中基质效应；*cis*-DCCA与*cis*-DBCA分别为102.4%和90.4%，属于低基质效应。文献表明，2-PBA与3-PBA结构相似，但2-PBA在常规拟除虫菊酯暴露中占比极低，主要由特定结构拟除虫菊酯或副产物代谢产生^［24，25］^，因此常被用作非同位素内标进行基质校正，具有经济可行的优势。综合考虑基质效应特点与校正需求，本研究选用2-PBA作为内标，并采用工作曲线法进行基质校正与定量分析。

#### 2.4.2 线性范围、检出限与定量限

将系列标准工作液与样品进行相同前处理后测定，以质量浓度为横坐标（*X*），定量离子与内标的峰面积比值为纵坐标（*Y*），拟合得曲线回归方程，见表2。参考GBZ/T 210.5-2008，以标准差法进行最低检出浓度实验：连续测定10次低浓度加标样品，计算测定的平均值和标准差（SD），以3倍SD×测定平均值/理论加标浓度，作为方法的检出限（LOD），以10倍SD×测定平均值/理论加标浓度，作为方法的定量限（LOQ）。结果表明，5种分析物的线性情况良好，相关系数（*r*）>0.995，方法的检出限范围为0.13~1.32 ng/mL，定量限范围为0.44~4.39 ng/mL。

**表2:** 5种分析物的线性方程、相关系数、检出限和定量限

Compound	Linear equations	*r*	LOD/（ng/mL）	LOQ/（ng/mL）
*trans*-DCCA	*Y*=3.68×10^-2^ *X*+4.01×10^-3^	0.999	0.18	0.61
*cis*-DCCA	*Y*=1.31×10^-3^ *X*-5.19×10^-4^	0.999	1.32	4.39
3-PBA	*Y*=1.92*X*+1.60	0.999	0.20	0.67
4F-3PBA	*Y*=2.27*X*+2.98×10^-2^	0.999	0.13	0.44
*cis*-DBCA	*Y*=1.55×10^-3^ *X*+1.28×10^-3^	0.997	1.21	4.03

*Y*： quantitative ion peak area/internal standard peak area； *X*： mass concentration of target compound， ng/mL.

#### 2.4.3 准确度和精密度

参考GBZ 210.5-2008，根据标准曲线线性范围，以非接触者的混合尿样为基质配制20、50、80 ng/mL 3个水平的加标样品，每个浓度6份，同时测定样品本底值，以样品加标回收法测定平均加标回收率作为方法准确度，以测定结果的相对标准偏差（RSD）作为方法的批内精密度；对3个浓度的加标样品，在3天内进行6次重复测定，计算6组数据的RSD作为方法的批间精密度，结果见表3，方法准确度为91.0%~102.0%，批内精密度为1.1%~8.1%，批间精密度为1.1%~4.6%，均<10%。

**表3:** 5种分析物在3个水平下的回收率和相对标准偏差（*n*=6）

Compound	Background/（ng/mL）	Spiked/（ng/mL）	Found/（ng/mL）	Recovery/%	Intra-batch RSD/%	Inter-batch RSD/%
*trans*-DCCA	-	20	20.4±0.3	102.0	1.4	2.3
	-	50	47.8±1.7	95.7	3.5	1.8
	-	80	79.7±1.9	99.6	2.4	1.9
*cis*-DCCA	-	20	18.7±0.6	93.6	3.5	3.3
	-	50	45.5±3.4	91.0	7.4	2.2
	-	80	75.7±4.1	94.6	5.4	1.4
3-PBA	-	20	20.3±0.2	101.4	1.1	2.3
	-	50	48.5±1.2	97.0	2.5	1.8
	-	80	78.1±1.3	97.6	1.7	1.1
4F-3PBA	-	20	20.2±0.3	101.2	1.4	2.2
	-	50	48.9±1.6	97.7	3.3	2.4
	-	80	80.4±1.0	100.5	1.3	1.8
*cis*-DBCA	3.1	20	22.2±1.5	95.5	8.1	4.6
	3.1	50	50.2±2.9	94.1	6.1	3.2
	3.1	80	83.7±2.3	100.7	2.8	4.2

-： not detected.

#### 2.4.4 稳定性实验

为评估目标分析物的保存稳定性，本研究采用非接触者混合尿液为基质，制备50 ng/mL加标样品，并于配制当日测定其初始浓度。样品分装后分别置于室温、4 ℃及-20 ℃条件下保存，每个温度设12份样本。其中A组（6份）于前处理当天添加内标，B组（6份）于分装时即添加内标，分别于第3天和第7天测定浓度并计算下降率（表4）。结果分析表明，A组数据主要反映了前处理等方法流程引入的波动，B组则在A组基础上进一步反映保存过程中分析物的稳定性变化。采用内标法计算时，以分析物与内标峰面积比值为纵坐标，理论上A组的下降率应高于B组。整体结果显示，目标分析物在室温下的下降率最高，可能与微生物分解有关；-20 ℃保存样本经冻融处理，下降率波动大于4 ℃条件。在4 ℃保存7天后，A、B两组样本下降率均低于10%，说明5种拟除虫菊酯代谢物在保存期间含量虽有所下降，但在4 ℃条件下可至少稳定保存7天。

**表4:** 5种分析物在不同保存条件下的下降率（*n*=3）

Compound	Temperature/℃	Decline rates/%			
		3 d （A）	3 d （B）	7 d （A）	7 d （B）
*trans*-DCCA	-20	5.3	0.4	8.5	8.8
	4	2.5	3.3	2.4	6.3
	RT	7.3	8.0	14.4	6.0
*cis*-DCCA	-20	6.0	4.5	8.3	4.1
	4	6.3	0.7	2.5	4.8
	RT	12.1	5.2	10.5	13.3
3-PBA	-20	6.1	1.2	3.1	4.3
	4	1.4	4.1	1.8	1.2
	RT	6.1	4.7	8.9	1.1
4F-3PBA	-20	4.6	3.3	3.3	4.7
	4	0.3	3.1	1.8	1.5
	RT	5.9	4.4	8.7	2.5
*cis*-DBCA	-20	15.0	0.8	3.3	0.9
	4	5.9	7.3	0.9	7.1
	RT	6.8	4.3	0.2	4.4

RT： room temperature； A： group with IS added on the day of pretreatment； B： group with IS added at the time of sample aliquoting.

#### 2.4.5 实际样品

收集办公室文职人员和未接触拟除虫菊酯的实验人员尿液样本共计18份，按照本方法测定5种拟除虫菊酯代谢产物含量，其中*cis*-DCCA和4F-3PBA均未检出，*trans*-DCCA有2人检出，高于定量限1人，含量0.87 ng/mL；*cis*-DBCA有4人检出，含量均低于定量限；3-PBA有16人检出，检出率88.9%，高于定量限8人，含量范围为0.69~1.59 ng/mL，该结果与国内外文献拟除虫菊酯代谢产物在人体含量筛查的研究中对3-PBA的检出率和检出范围基本一致^［24，26，27］^，导致普通人群3-PBA水平升高的因素主要包括饮食暴露以及家庭和农业除虫菊酯类农药的施用。

## 3 结论

本研究建立了尿中5种拟除虫菊酯代谢产物测定的超高效液相色谱-串联质谱分析方法，样品经盐酸水解，以乙酸乙酯萃取，经蒸发复溶后上机测定，以工作曲线内标法定量，样本前处理简便高效，成本低，且仪器分析程序时间较短，有利于方法的推广应用，方法的检出限低，准确度高且精密度好，可同时准确测定尿中5种拟除虫菊酯代谢产物含量，可为拟除虫菊酯接触人群，尤其是一般居民、孕妇、儿童等敏感群体及职业暴露监测评估研究提供技术参考。
